# Domain-specific Physical Activity and the Risk of All-cause Mortality Among Middle-aged and Older Adults in Taiwan: A Prospective Cohort Study

**DOI:** 10.2188/jea.JE20220105

**Published:** 2023-11-05

**Authors:** Yu-Tai Liu, Yung Liao, Ming-Chun Hsueh, Hsin-Yen Yen, Jong-Hwan Park, Jae Hyeok Chang

**Affiliations:** 1 Department of Physical Education and Sport Sciences, National Taiwan Normal University, Taipei, Taiwan; 2 Graduate Institute of Sport, Leisure and Hospitality Management, National Taiwan Normal University, Taipei, Taiwan; 3 Graduate Institute of Sport Pedagogy, University of Taipei, Taipei, Taiwan; 4 School of Gerontology and Long-Term Care, College of Nursing, Taipei Medical University, Taipei, Taiwan; 5 Health Behaviors & Disease Prevention Research Group, Biomedical Research Institute, Pusan National University Hospital, Busan, South Korea; 6 Department of Rehabilitation Medicine, School of Medicine, Pusan National University, Busan, South Korea

**Keywords:** all-cause mortality, leisure-time physical activity, household physical activity, cohort study

## Abstract

**Background:**

The impact of meeting leisure-time physical activity (LTPA) recommendations and household physical activity (HPA) on all-cause mortality in the Taiwanese population is unclear. We aimed to investigate the relationship between sufficient LTPA and all-cause mortality in middle-aged and older Taiwanese adults and the role of HPA in those with insufficient LTPA.

**Methods:**

This nationwide prospective cohort study included 4,960 participants aged ≥50 years from the Taiwan Longitudinal Study in Aging (TLSA) survey. Physical activity patterns were assessed in 2003 and then followed up until 2015 for mortality through the National Death Registration Record. Cox proportional hazards regression was conducted to evaluate hazard ratios (HRs) and 95% confidence intervals (CIs) for all-cause mortality.

**Results:**

Of the 4,960 participants, 1,712 died of all-cause mortality. Compared to those who had insufficient LTPA, participants who engaged in sufficient LTPA showed a significantly lower risk of all-cause mortality (HR = 0.84, 95% CI, 0.73–0.97). For those with insufficient LTPA, HPA also had a significantly reduced risk of all-cause mortality (HR = 0.85, 95% CI, 0.75–0.96) among general population. Similar associations were observed in subsequent sensitivity analyses. The subgroup analysis showed that the relationship between HPA and reduced mortality risk was only found in the women with insufficient LTPA group.

**Conclusion:**

This study confirmed that sufficient LTPA is associated with a lower risk of all-cause mortality. If sufficient LTPA cannot be performed, additional HPA is related to lower mortality.

## INTRODUCTION

Over the last few decades, Taiwan has become an aged society due to its high medical standards and low birth rate. The proportion of adults aged ≥65 years increased from 7% to 14% between 1993 and 2018. In the near future, the proportion of older adults is estimated to exceed 20%.^1^ It is well known that the development of disease and disability that comes with aging harms life expectancy. To overcome health challenges, extending the average lifespan by having good health has gradually been emphasized. There is growing evidence that middle-aged and older adults adopting a healthy lifestyle, such as engaging in sufficient physical activity (PA), may improve their healthy life expectancy.^2^^,^^3^

Contrastingly, insufficient PA can lead to an unhealthy condition and can come with high health and financial costs.^4^ The World Health Organization’s (WHO) most recent report indicated that globally, 23% of adults were physically inactive.^3^ In the United States, 27.5% of adults aged ≥50 years did not have sufficient PA.^5^ The rate of physical inactivity ranged from 4.9–29% in European countries.^6^ In Taiwan, only 33% of people engaged in regular PA in 2020.^7^ Lack of PA has emerged as a major public health problem globally.^8^^,^^9^ Subsequently, WHO stated insufficient PA as one of the leading risk factors for worldwide mortality.^10^

There are several official recommendations and guidelines worldwide to promote PA. Studies have shown the benefit of PA in lifespan prolongation in Taiwan.^11^^–^^15^ However, limited studies exist on whether meeting leisure-time physical activity (LTPA) recommendations can reduce the risks of all-cause mortality among Taiwanese middle-aged and older adults.

PA is commonly undertaken in four domains: leisure time, household, occupational, and transportation.^10^ These different domains are varied in time-use composition.^16^ LTPA could be largely carried out during leisure time. Household physical activity (HPA) may be an inherent aspect in many people’s daily lives. Interestingly, it has been found that HPA is able to compensate for a low level of LTPA.^17^ Therefore, the impact of the LTPA and HPA domains on the risk of all-cause mortality is an important area of study. Since HPA is already an available tool for many, it is worthwhile to investigate its role on mortality risk among people with insufficient LTPA, and whether gender difference existed in the HPA results.

To investigate the long-term associations between PA and all-cause mortality in middle-aged and older adults, this study was conducted based on a prospective cohort study design. First, this study examined the association between meeting the LTPA recommendations and all-cause mortality. Additionally, this study aimed to evaluate the relationship between HPA and all-cause mortality in populations with insufficient LTPA. Two hypotheses were formulated in this study: (1) sufficient LTPA among adults is associated with a lower risk of all-cause mortality compared with those who do not have sufficient LTPA; and (2) for those with insufficient LTPA, engaging in HPA was related to a reduced risk of all-cause mortality compared with those who did not engage in it.

## METHODS

### Study population and study design

**Figure 1.**
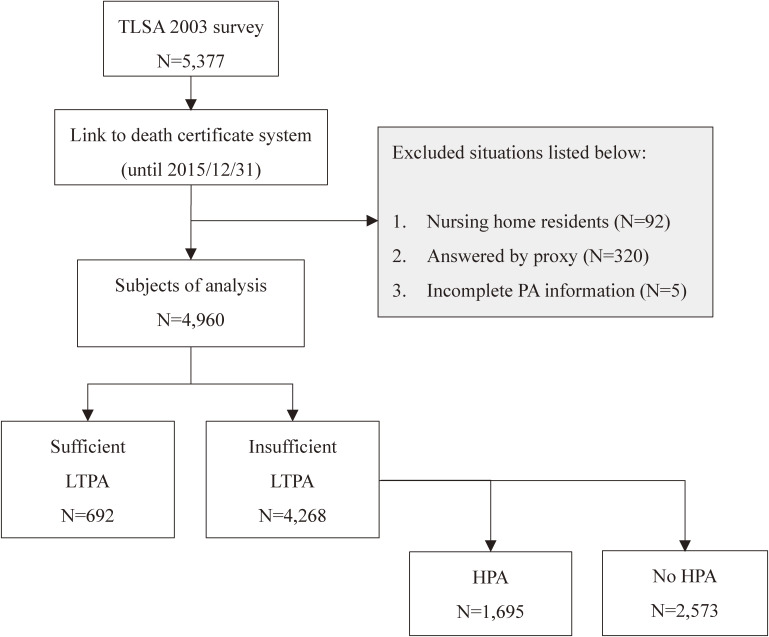
Flow chart of inclusion and exclusion. HPA, household physical activity; LTPA, leisure-time physical activity.

The data used here were obtained from the Taiwan Longitudinal Study on Aging (TLSA), a national survey of a representative Taiwanese adult sample conducted by the Bureau of Health Promotion, Ministry of Health and Welfare of Taiwan. The TLSA was initiated in 1989 and used a complex survey design with a stratified multistage equal probability sampling method to represent the distribution of Taiwanese adults. Trained interviewers delivered the TLSA questionnaire via in-home or in-person interviews to obtain personal and health-related information from each participant. The surveys were conducted in 1993, 1996, 1999, 2003, 2007, and 2011. All data were linked to national death registrations to record deaths or follow-up loss between waves. The first cohort of 4,049 adults aged ≥60 years completed the survey in 1989. The second and third cohorts of supplemental samples with participants aged 50–66 years were included in the 1996 and 2003 surveys, with 2,462 and 1,599 adults, respectively. The response rate in each survey wave was high, ranging from 79.1–92.1%. This study included 5,377 participants from the 2003 survey that covered all three cohorts of adults to determine the longitudinal association between PA and all-cause mortality. To ensure the robustness of the results, participants who lived in care institutions or answered by proxy, as well as those who provided incomplete PA information, were excluded. The remaining 4,960 participants were used as baseline data to examine the risk of all-cause mortality from 2003–2015. The flowchart of inclusion and exclusion is illustrated in Figure 1. The study was approved by the National Taiwan Normal University Ethics Committee with a waiver of informed consent because deidentified data were used (approval document number 202103HM011).

### All-cause mortality

All-cause mortality was the only dependent variable measured in survival years from 2003 to 2015 using Taiwan’s National Death Registration Record, which contains survival status and date of death. Participants’ national identification numbers were used to link the data from the two databases during the follow-up surveys.

### Physical activity

The volume and domains of PA (LTPA and HPA) were assessed using the 2003 TLSA questionnaire. LTPA exposure was measured by asking about its frequency (0, ≤2, 3–5, or ≥6 times per week) and duration (<15 min, 15–30 min, or >30 min). The intensity was classified based on panting level after PA with light intensity, moderate intensity, and vigorous intensity. Following the latest WHO 2020 guidelines on physical activity, the participants of this study were categorized into two groups: meeting the current recommendation (150 minutes of moderate-intensity activity each week or 75 minutes of vigorous-intensity activity) and not meeting the current recommendation (<150 minutes of light- to moderate-intensity activity each week and <75 minutes of vigorous-intensity activity). The question on HPA was the following: “Who is doing most of the lighter and necessary work in your house?” These include cooking, washing, sweeping, cleaning, washing dishes, or buying vegetables. All participants were classified as being either involved in HPA (do the work yourself) or not involved (others do the work). Patterns in PA domains refer to leisure time and household groups in the 2003 survey. LTPA was classified as (1) sufficient LTPA, meeting the LTPA recommendation; and (2) insufficient LTPA, not meeting the LTPA recommendation. HPA was classified as: (1) HPA: engaging in household work or (2) no HPA: not engaging in household work.

### Covariates

Several baseline characteristics, such as basic personal variables, discrete personal variables, lifestyle factors, and chronic health conditions, were drawn from the 2003 baseline survey. These variables were analyzed as covariates to minimize confounding factors. Basic personal variables included sex and age. Discrete personal variables included educational level (without formal education, with elementary school, junior to senior high school, and college degree or above), marital status (married or otherwise), area (rural, suburban, or urban), and economic satisfaction (satisfied, fair, or dissatisfied). Lifestyle factors included body mass index (BMI; underweight, normal weight, overweight, and obese), smoking status, and alcohol intake. Smoking status was classified as current, former, and never smoker by the questions: “Do you still smoke?” and “Have you ever smoked?” Alcohol intake was defined as lifetime abstainer, infrequent, light-to-moderate, and heavy drinkers, according to self-reported drinking frequency (times per week, month, and year). Chronic health conditions (hypertension, diabetes mellitus, heart disease, stroke, and cancer) were defined by the participants’ responses about whether the clinician informed them that they had chronic illnesses.

### Statistical analysis

The current study used the chi-square test to assess differences in baseline characteristics of all the participants across the groups in LTPA and HPA patterns. To investigate the role of PA on all-cause mortality in study population, a Cox proportional hazards regression model was conducted, and hazard ratios (HRs) with 95% confidence intervals (CIs) were presented. Each participant’s follow-up time was accumulated from the 2003 survey up to death or to the end of the study period. Three models with different groups of covariates were developed to test the potential confounding effects on survival analyses. Model 1 was adjusted for sex and age; model 2 was additionally adjusted for discrete personal variables (educational level, marital status, area, economic satisfaction, BMI, smoking status, and alcohol intake). Model 3 additionally included chronic health conditions (hypertension, diabetes, heart disease, stroke, and cancer). Considering the potential bias derived from the selected participants, this study performed three sensitivity analyses. The first sensitivity analysis was conducted by excluding very old adults (≥85 years old) and premature mortality (deceased during the first year of follow-up). The second sensitivity analysis was performed by excluding adults with any chronic health conditions at baseline. The third sensitivity analysis was conducted to test the consistency of the LTPA and mortality results. A 1:1 exact matching on sex and age was used to ensure that the insufficient and sufficient LTPA groups were as similar as possible. To clarify interaction, survival analyses with the combination of LTPA and HPA were performed. The roles of HPA in overall and sufficient LTPA participants were also examined. As a potential sex difference in mortality risk might exist among participants with HPA, subgroup analyses were performed to identify the sex difference in results with HPA and combined patterns. The flowchart of the inclusion and exclusion of sensitivity is illustrated in eFigure 1, eFigure 2, and eFigure 3. All the analyses were performed using SAS v9.4 (SAS Institute, Cary, NC, USA). Two-tailed *P* values less than 0.05 were considered statistically significant.

## RESULTS

A total of 4,960 eligible participants were selected from the 2003 survey, of which 51.53% were men. Of the 4,960 participants, 13.95% (*n* = 692) engaged in sufficient LTPA and 86.05% (*n* = 4,268) in insufficient LTPA. The baseline characteristics of the participants are described in Table 1. The proportion of adults of both sexes who engaged in LTPA decreased with age. There were significant differences in the baseline characteristics across the two activity groups, including sex, educational level, living area, economic satisfaction, smoking status, alcohol intake, and chronic diseases. Compared with the insufficient LTPA group, those who engaged in sufficient LTPA were more inclined to be male, be younger, have a high education level, be married, be urban residents, report economic satisfaction, and report fewer chronic conditions. Among those who reported insufficient LTPA (86.05%), the proportions of HPA were as follows: 39.71% (*n* = 1,695) engaged in HPA and 60.29% (*n* = 2,573) did not engage in HPA. The detailed baseline characteristics of HPA participants are presented in eTable 1.

**Table 1.** Baseline characteristics of participants by the pattern of LTPA and TLSA, 2003

Characteristics	Type of LTPA			*P*-value

	Sufficient LTPA, *N* (%)	Insufficient LTPA, *N* (%)	Total, *N*	
	692 (13.95)	4,268 (86.05)	4,960	
Sex				

Men	410 (59.25)	21,46 (50.28)	2,556	<0.001
Women	282 (40.75)	2,122 (49.72)	2,404	

Age group, years				

50–64	344 (49.71)	2,214 (51.87)	2,558	0.130
65–74	174 (25.14)	934 (21.88)	1,108	
75–84	161 (23.27)	996 (23.34)	1,157	
≥*85*	13 (1.88)	124 (2.91)	137	

Educational level				

Without formal education	142 (20.52)	1,095 (25.66)	1,237	0.010
Elementary school	300 (43.35)	1,835 (42.99)	2,135	
Junior to senior high school	173 (25)	959 (22.47)	1,132	
College degree and above	77 (11.13)	379 (8.88)	456	

Marital status				

Married	509 (73.55)	3,048 (71.42)	3,557	0.246
Unmarried	183 (26.45)	1,220 (28.58)	1,403	

Area				

Rural	217 (31.36)	1,441 (33.76)	1,658	0.023
Suburban	139 (20.09)	1,001 (23.45)	1,140	
Urban	335 (48.41)	1,812 (42.46)	2,147	
^*^missing	1 (0.14)	14 (0.33)	15	

Economic satisfaction				

Satisfied	291 (42.05)	1,614 (37.82)	1,905	0.013
Fair	243 (35.12)	1,460 (34.21)	1,703	
Dissatisfied	158 (22.83)	1,194 (27.98)	1,352	

BMI, kg/m^2^				

Underweight	27 (3.9)	211 (4.94)	238	0.112
Normal weight	324 (46.82)	1,982 (46.44)	2,306	
Overweight	193 (27.89)	1,161 (27.2)	1,354	
Obesity	134 (19.36)	754 (17.67)	888	
^*^missing	14 (2.02)	160 (3.75)	174	

Smoking status				

Never	378 (54.62)	2,672 (62.61)	3,050	<0.001
Former	152 (21.97)	653 (15.3)	805	
Current	162 (23.41)	943 (22.09)	1,105	

Alcohol intake				

Lifetime abstainer	468 (67.63)	2,996 (70.2)	3,464	0.257
Infrequent	80 (11.56)	393 (9.21)	473	
Light to moderate	97 (14.02)	596 (13.96)	693	
Heavy	47 (6.79)	283 (6.63)	330	

Hypertension				

No	428 (61.85)	2,916 (68.32)	3,344	0.001
Yes	264 (38.15)	1,352 (31.68)	1,616	

Diabetes				

No	596 (86.13)	3,681 (86.25)	4,277	0.933
Yes	96 (13.87)	587 (13.75)	683	

Heart disease				

No	559 (80.78)	3,592 (84.16)	4,151	0.026
Yes	133 (19.22)	676 (15.84)	809	

Stroke				

No	663 (95.81)	4,089 (95.81)	4,752	0.997
Yes	29 (4.19)	179 (4.19)	208	

Cancer				

No	676 (97.69)	4,143 (97.07)	4,819	0.365
Yes	16 (2.31)	125 (2.93)	141	

LTPA, leisure-time physical activity; TLSA, Taiwan Longitudinal Study on Aging.

The mean follow-up duration of the study endpoint was 12 years. A total of 1,712 adults died from all-causes. The Cox regression model illustrated that the sufficient LTPA group was associated with a significantly lower risk of all-cause mortality than the insufficient LTPA group. The mortality incidence rate (per 1,000 adults) for sufficient LTPA adults was 30.4, and it was 34.5 for those with insufficient LTPA. After adjustment for sex and age (model 1), in comparison with the insufficient LTPA group, the risk of all-cause mortality was reduced by 20% (HR 0.80; 95% CI, 0.69–0.92) in those with sufficient LTPA. In contrast to the insufficient LTPA group, the sufficient LTPA group had a reduced risk of all-cause mortality by 17% (HR 0.83; 95% CI, 0.72–0.96) after adjusting for sex, age, and discrete personal variables and lifestyle factors (model 2). In the fully adjusted model (model 3), the sufficient LTPA group had a significantly reduced risk of all-cause mortality by 16% (HR 0.84; 95% CI, 0.73–0.97). The results of the survival analysis for LTPA are shown in Table 2.

**Table 2.** Association between LTPA pattern and subsequent risk of all-cause mortality (*N* = 4,960)

Group	*N*	Number of deaths	Person-years	Incidence rate (/10^3^ person-year)	Hazard ratio (95% CI)	*P*-value	Model
Insufficient LTPA	4,268	1,493	43,309	34.5	1 [Reference]		
Sufficient LTPA	692	219	7,198	30.4	0.80 (0.69–0.92)	0.002	Model 1^a^
					0.83 (0.72–0.96)	0.010	Model 2^b^
					0.84 (0.73–0.97)	0.016	Model 3^c^

CI, confidence interval; LTPA, leisure-time physical activity.

^a^Model 1: Adjusted for sex and age.

^b^Model 2: model 1 + educational, marital, area, economic satisfaction, body mass index, smoking status, and alcohol intake.

^c^Model 3: model 2 + chronic health conditions.

Overall, sufficient LTPA was associated with a lower risk of all-cause mortality among adults. For adults with insufficient LTPA, engaging in HPA was associated with reduced all-cause mortality. The all-cause mortality rate (per 1,000 adults) for participants with HPA was 23.9, and for those with no HPA, it was 42.1. The adjusted HRs for all-cause mortality were 0.78 (95% CI, 0.69–0.88), 0.78 (95% CI, 0.69–0.88), and 0.85 (95% CI, 0.75–0.96) in model 1, model 2, and model 3, respectively. The results of the survival analysis in the HPA are shown in Table 3. After stratifying the population by sex, the participation rate in HPA varied between men (16.21%) and women (63.47%). Subgroup analysis (see Table 4) showed that all-cause mortality risk decreased with HPA in women (HR 0.72; 95% CI, 0.61–0.86) but slightly increased with HPA in men (HR 1.19; 95% CI, 0.99–1.44).

**Table 3.** Association between HPA pattern and subsequent risk of all-cause mortality among people with insufficient LTPA (*N* = 4,268)

Group	*N*	Number of deaths	Person-years	Incidence rate (/10^3^ person-year)	Hazard ratio (95% CI)	*P*-value	Model
No HPA	2,573	1,057	25,085	42.1	1 [Reference]		
HPA	1,695	436	18,224	23.9	0.78 (0.69–0.88)	<0.001	Model 1^a^
					0.78 (0.69–0.88)	<0.001	Model 2^b^
					0.85 (0.75–0.96)	0.008	Model 3^c^

CI, confidence interval; HPA, household physical activity; LTPA, leisure-time physical activity.

^a^Model 1: Adjusted for sex and age.

^b^Model 2: model 1 + educational, marital, area, economic satisfaction, body mass index, smoking status, and alcohol intake.

^c^Model 3: model 2 + chronic health conditions.

**Table 4.** Subgroup analysis^a^ of association between HPA pattern and subsequent risk of all-cause mortality among people with insufficient LTPA

	Type of HPA		

	HR (95% CI)		

	No HPA	HPA	*P*-value
Overall	1 [Reference]	0.85 (0.75–0.96)	0.008

Subgroup			

Men	1 [Reference]	1.19 (0.99–1.44)	0.056
Women	1 [Reference]	0.72 (0.61–0.86)	<0.001

CI, confidence interval; HPA, household physical activity; HR, hazard ratio; LTPA, leisure-time physical activity.

^a^Adjusted for sex, age, education, marital status, area, economic satisfaction, body mass index, smoking status, and alcohol intake, and chronic health conditions.

Three sensitivity analyses were conducted to confirm these findings. First, analysis was performed excluding adults aged ≥85 years (*N* = 137) and mortality events occurred in the first year (*N* = 15). The significantly lower risk of all-cause mortality associated with sufficient LTPA and HPA was consistent between the primary and sensitivity analyses. Second, the exclusion of adults with any chronic health conditions at baseline also showed a lower risk of all-cause mortality among adults with sufficient LTPA and HPA. The overall survival results of the first and second sensitivity analysis are shown in Table 5 and Table 6, respectively. Third, among LTPA participants, the sufficient and insufficient groups were 1:1 matched for sex and age. The results in Table 7 show that the association between LTPA and all-cause mortality was consistent with that of the main analyses. The baseline characteristics in sensitivity analyses are presented in eTable 2, eTable 3, eTable 4, eTable 5, and eTable 6. No interaction between LTPA and HPA for all-cause mortality was found in either interaction test and survival analyses with combined patterns (see eTable 7, eTable 8, and eTable 9). The results of HPA in the overall and sufficient LTPA population are shown in eTable 10 and eTable 11.

**Table 5.** Association between PA pattern and subsequent risk of all-cause mortality: sensitivity analysis excluding very old adults (>85 years old) and premature mortality

Group	*N*	Number of deaths	Hazard ratio (95% CI)	*P*-value	Model
LTPA group (*N* = 4,808)					

Insufficient LTPA	4,132	1,367	1 [Reference]		
Sufficient LTPA	676	205	0.8 (0.69–0.93)	0.003	Model 1^a^
			0.83 (0.71–0.96)	0.011	Model 2^b^
			0.83 (0.72–0.97)	0.017	Model 3^c^

HPA group (*N* = 4,132)					

No HPA	2,458	950	1 [Reference]		
HPA	1,674	417	0.79 (0.69–0.89)	<0.001	Model 1^a^
			0.79 (0.70–0.89)	<0.001	Model 2^b^
			0.86 (0.76–0.98)	0.023	Model 3^c^

CI, confidence interval; HPA, household physical activity; LTPA, leisure-time physical activity; PA, physical activity.

^a^Model 1: Adjusted for sex and age.

^b^Model 2: model 1 + education, marital status, area, economic satisfaction, body mass index, smoking status, and alcohol intake.

^c^Model 3: model 2 + chronic health conditions.

**Table 6.** Association between PA pattern and subsequent risk of all-cause: sensitivity analysis excluding adults with chronic health conditions

Group	*N*	Number of deaths	Hazard ratio (95% CI)	*P*-value	Model
LTPA group (*N* = 2,661)					

Insufficient LTPA	2,339	585	1 [Reference]		
Sufficient LTPA	322	62	0.67 (0.51–0.87)	0.003	Model 1^a^
			0.71 (0.54–0.92)	0.011	Model 2^b^

HPA group (*N* = 2,339)					

No HPA	1,367	404	1 [Reference]		
HPA	972	181	0.99 (0.82–1.21)	0.954	Model 1^a^
			0.95 (0.78–1.15)	0.573	Model 2^b^

CI, confidence interval; HPA, household physical activity; LTPA, leisure-time physical activity; PA, physical activity.

^a^Model 1: Adjusted for sex and age.

^b^Model 2: model 1 + education, marital status, area, economic satisfaction, body mass index, smoking status, and alcohol intake.

**Table 7.** Association between matched LTPA pattern and subsequent risk of all-cause mortality: sensitivity analysis by matching (*N* = 1,384)

Group	*N*	Number of deaths	Person-years	Incidence rate (/10^3^ person-year)	Hazard ratio (95% CI)	*P*-value	Model
Insufficient LTPA	692	260	6,962	0.0373	1 [Reference]		
Sufficient LTPA	692	219	7,198	0.0304	0.77 (0.64–0.92)	0.005	Model 1^a^
					0.79 (0.66–0.95)	0.011	Model 2^b^
					0.78 (0.65–0.94)	0.009	Model 3^c^

CI, confidence interval; LTPA, leisure-time physical activity.

^a^Model 1: Adjusted for sex and age.

^b^Model 2: model 1 + education, marital status, area, economic satisfaction, body mass index, smoking status, and alcohol intake.

^c^Model 3: model 2 + chronic health conditions.

## DISCUSSION

In this prospective cohort study of middle-aged and older Taiwanese adults, the findings support the hypothesis that those who achieved sufficient LTPA have a lower risk of all-cause mortality. These findings provide further evidence to support the amount of LTPA, consistent with the recommendations for lower mortality. Furthermore, for those who did not achieve sufficient LTPA, engaging in HPA also showed a reduced risk of all-cause mortality. To our knowledge, this is the first study on HPA and mortality among adults with insufficient LTPA. The study findings highlight the practical importance of prescribing specific PA domains to prolong life expectancy.

Many studies have reported on the impact of LTPA exposure on all-cause mortality. The findings of this study add to the accumulating evidence highlighting that meeting official PA recommendations has a significant influence on all-cause mortality. A study of PA and mortality in American adults aged ≥18 years showed that meeting the 2008 Physical Activity Guidelines was inversely associated with mortality risk.^18^ The Pooled Analysis Study of six cohorts of United States adults aged 21–98 years also supported that performing sufficient moderate or vigorous intensity activities has longevity benefits.^19^ Two studies found that older adults aged ≥65 years who engaged in sufficient LTPA had lower risk of all-cause mortality.^20^^,^^21^ However, from a methodological point of view, inactive participants were usually analyzed as a reference group among most studies. Few studies have examined mortality risk by comparing participants meeting recommendations to those who did not meet the recommendations. In this study, inactive and active participants were not analyzed together. The present findings are similar to those of two recent studies. A cohort study investigated 479,856 United States adults ≥18 years old,^22^ and a pooled analysis included data from 11 population cohorts^23^: both studies showed that adults performing sufficient LTPA had a significantly lower risk of death than those who did not.

Data from surveys of 168 countries showed that the global prevalence of insufficient PA was 27.5% in adults aged ≥18 years.^24^ For those who did not achieve sufficient PA, evidence has proven that engaging in low-volume PA or doing domain-specific PA could also bring additional health benefits.^14^^,^^25^ Preliminary research indicated that a reduction in sedentary time by expanding bouts of light-intensity PA is achievable in older populations. Among the domains of PA, the HPA makes an essential contribution to total PA.^25^ Previous studies have investigated the association between HPA and mortality. A recent study reported that HPA could delay all-cause mortality in Korean middle-aged adults.^26^ A cohort study in adults aged 25–74 years who engaged in HPA also showed an 18% lower risk of all-cause mortality.^27^ For older adults with different domains of PA, HPA contributed to a significantly lower risk of death.^28^ Similarly, reduction in mortality risk was also reported in a study of Taiwanese older adults.^11^ However, previous studies detected an inverse association between HPA only in the general population.^11^^,^^26^^–^^28^ The association between HPA and all-cause mortality among those who engaged in insufficient LTPA was absent. This study is the first to investigate the role of HPA in populations who did not engage in sufficient LTPA.

In a subgroup analysis for HPA, the result of the men’s group was inconsistent with the overall population, women’s group, and previous research.^11^^,^^26^ Previous studies revealed that women tend to do more household chores than men, especially in couple relationships.^29^^,^^30^ A man who performs HPA in old age might not have a partner or might be living alone. Thus, a possible explanation for the sex difference in mortality risk is that men included in this study who did household chores had poor social support, which has been proven to be detrimental to health.^31^^,^^32^ Nevertheless, future research is needed to investigate the sex difference between HPA and all-cause mortality.

Several biological mechanisms may account for the reduced risk of all-cause mortality associated with sufficient LTPA and HPA.^33^ Possible mechanisms include boosting circulation, reducing serum lipid levels, improving insulin sensitivity, and strengthening the immune system. Additionally, LTPA and HPA positively influence the musculoskeletal system by involving multiple muscle groups. Sufficient strength can help sustain movement. HPA often involves smaller muscle groups and isometric contractions. Nevertheless, HPA plays an important role in survival benefits by reducing sedentariness and boosting overall PA.

As is common in observational studies, the survival benefits of PA can be attributed to the effects of residual confounding or reverse causation. Insufficient LTPA or absence of HPA might be a consequence of adverse health conditions at the time of the survey interview. To minimize potential bias, this study used robust approaches and performed sensitivity analyses. First, multiple regression was used to estimate the adjusted HRs by controlling for baseline covariates including personal characteristics, lifestyles, and chronic health conditions. Second, sensitivity analyses were performed to confirm the findings. And the results are similar to previous studies that conducted sensitivity analyses excluding the aging or low health status people.^34^^,^^35^ Additionally, a 1:1 exact matching for age and sex was used to analyze the association between LTPA and all-cause mortality.

This study captures useful domain-specific information with the self-report method and extracts meaningful findings. These findings have important implications in clinical practice, as they suggest that middle-aged and older adults could be protected from premature mortality by performing at least 150 minutes of moderate-intensity LTPA or 75 minutes of vigorous-intensity LTPA per week. Additionally, for those who are unable or unwilling to perform sufficient LTPA, the HPA could also play an important role in reducing premature mortality risk. This investigation emphasizes the necessity of engaging in sufficient LTPA and suggests that further recommendations should be broadened to promote more achievable PA goals, such as HPA, for the general population.

### Limitations

This study has some limitations. First, the information on PA was self-reported and lacked formal validation, which may have resulted in some misclassification. Compared with objective measurements, such as accelerometers, questionnaires are more liable for recall bias. Second, LTPA, HPA, and all other study variables were assessed only at the baseline of the follow-up. The patterns of lifestyle habits may have changed over time. This may have led to an inaccurate estimation of the protective effect of PA on mortality. Third, the HPA was determined only by asking a single dichotomous question. Information on the type, frequency, and intensity of the HPA was not assessed. There was a less detailed definition in the questionnaire to evaluate engagement in activities at home. Fourth, information on the severity of the reported health conditions was not available. Some of the survival benefits of PA might account for the lower severity in participants who were able to perform PA. Fifth, the difference in dose-response between domains was not clarified, since no objective dimension between LTPA and HPA was established in this study. Sixth, the cohort used in this study only consisted of middle-aged and older Taiwanese adults. This might limit the generalizability of the findings to different populations. Finally, because the survival benefit of HPA was only found in the insufficient LTPA population, future studies could be performed in a representative sufficient LTPA population.

### Conclusion

The current nationwide prospective cohort study confirmed that meeting the LTPA recommendations is associated with all-cause mortality. Additionally, this study showed that HPA could be independently beneficial for the life longevity of populations with insufficient LTPA, particularly in women. These findings provide vital information for individuals with insufficient PA, and that performing the recommended amount of LTPA or HPA is related to lowering risk of all-cause mortality. Given the significant benefits of PA, the main future effort is to promote an active lifestyle, including adherence to LTPA recommendations and participation in HPA.

## SUPPLEMENTARY MATERIAL

eFigure 1. Flow chart of inclusion and exclusion in 1st sensitivity analyses. HPA, household physical activity; LTPA, leisure-time physical activity.

eFigure 2. Flow chart of inclusion and exclusion in 2nd sensitivity analyses. HPA, household physical activity; LTPA, leisure-time physical activity,

eFigure 3. Flow chart of inclusion and exclusion in 3rd sensitivity analyses. LTPA, leisure-time physical activity.

eTable 1. Baseline characteristics of participants with insufficient LTPA by the patterns of HPA, and TLSA, 2003

eTable 2. Baseline characteristics of participants by the patterns of LTPA in 1^st^ sensitivity analysis, TLSA, 2003

eTable 3. Baseline characteristics of participants by the patterns of LTPA in 2^nd^ sensitivity analysis, TLSA, 2003

eTable 4. Baseline characteristics of matched participants by the patterns of LTPA in 3^rd^ sensitivity analysis, TLSA, 2003

eTable 5. Baseline characteristics of participants with insufficient LTPA by the patterns of HPA in 1^st^ sensitivity analysis, TLSA, 2003

eTable 6. Baseline characteristics of participants with insufficient LTPA by the patterns of HPA in 2^nd^ sensitivity analysis, TLSA, 2003

eTable 7. Wald Tests of HPA*LTPA Effect

eTable 8, Association of the patterns of combined LTPA and HPA with subsequent risk of all-cause mortality (N = 4,960)

eTable 9. Subgroup analysis of the association of the patterns of combined LTPA and HPA with subsequent risk of all-cause mortality

eTable 10. Association between HPA patterns and subsequent risk of all-cause mortality among overall participants (N = 4,960)

eTable 11. Association between HPA patterns and subsequent risk of all-cause mortality among people with sufficient LTPA (N = 692)
