# Supplementary material for: Domain-specific Physical Activity and the Risk of All-cause Mortality Among Middle-aged and Older Adults in Taiwan: A Prospective Cohort Study
Source: J Epidemiol. 2023 Nov 5;33(11):574–81. doi: 10.2188/jea.JE20220105 (PMC10518382; doi:10.2188/jea.JE20220105)
Supplement: Supplementary file 1 [file je-33-574-s001.pdf]

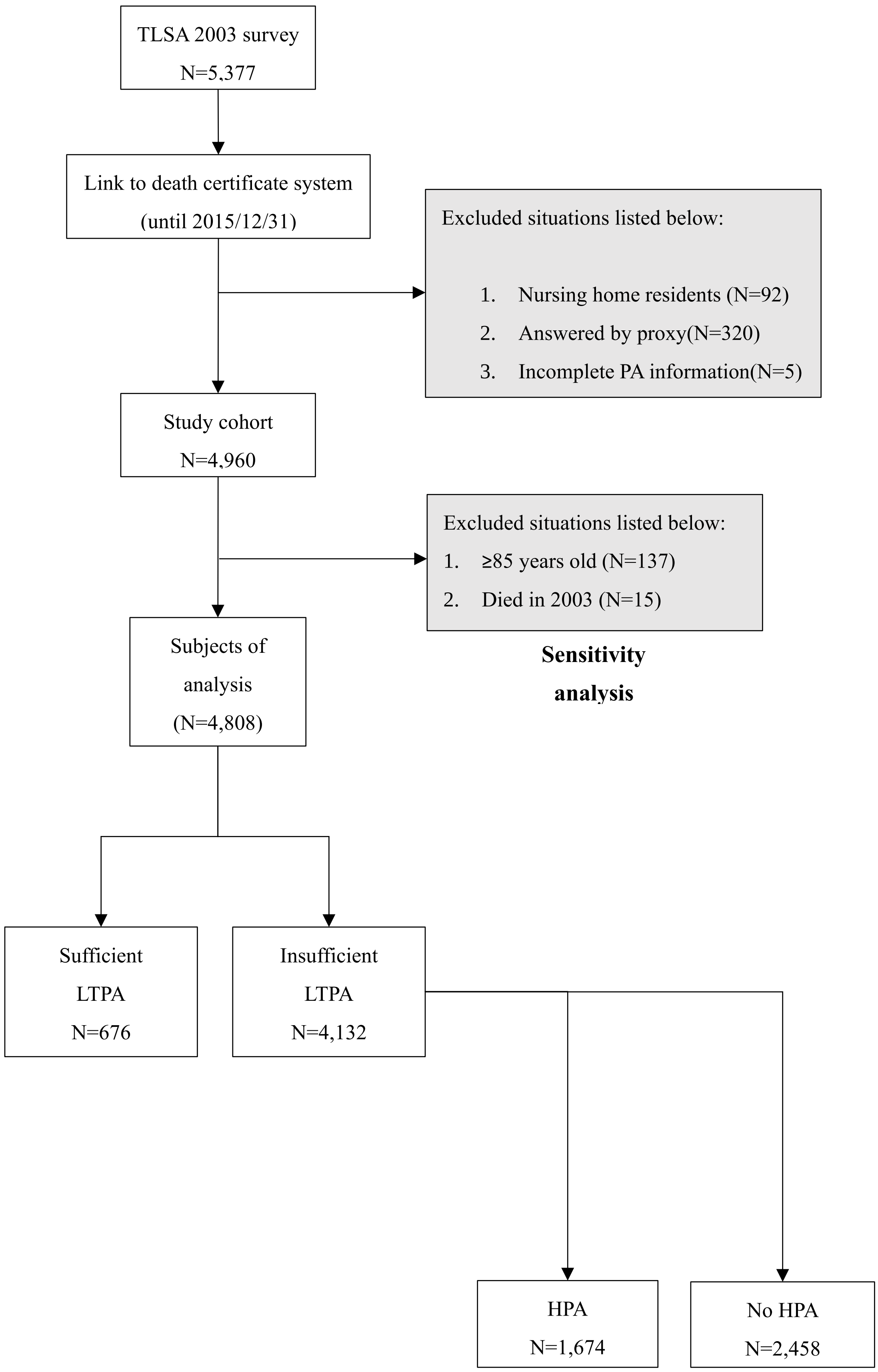

eFigure 1. Flow chart of inclusion and exclusion in 1st sensitivity analyses. HPA, household physical activity; LTPA, leisure-time physical activity.

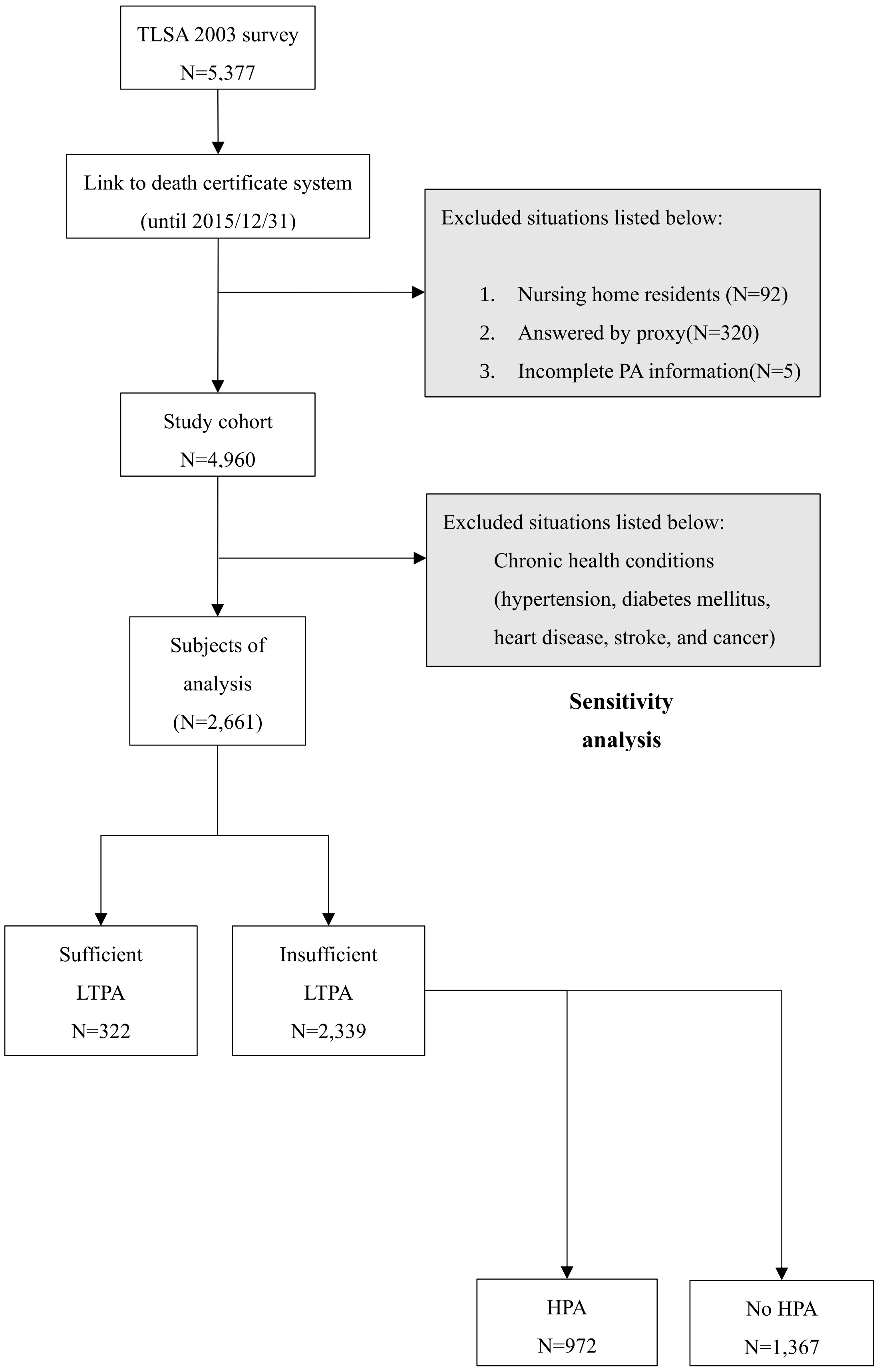

eFigure 2. Flow chart of inclusion and exclusion in 2nd sensitivity analyses. HPA, household physical activity; LTPA, leisure-time physical activity,

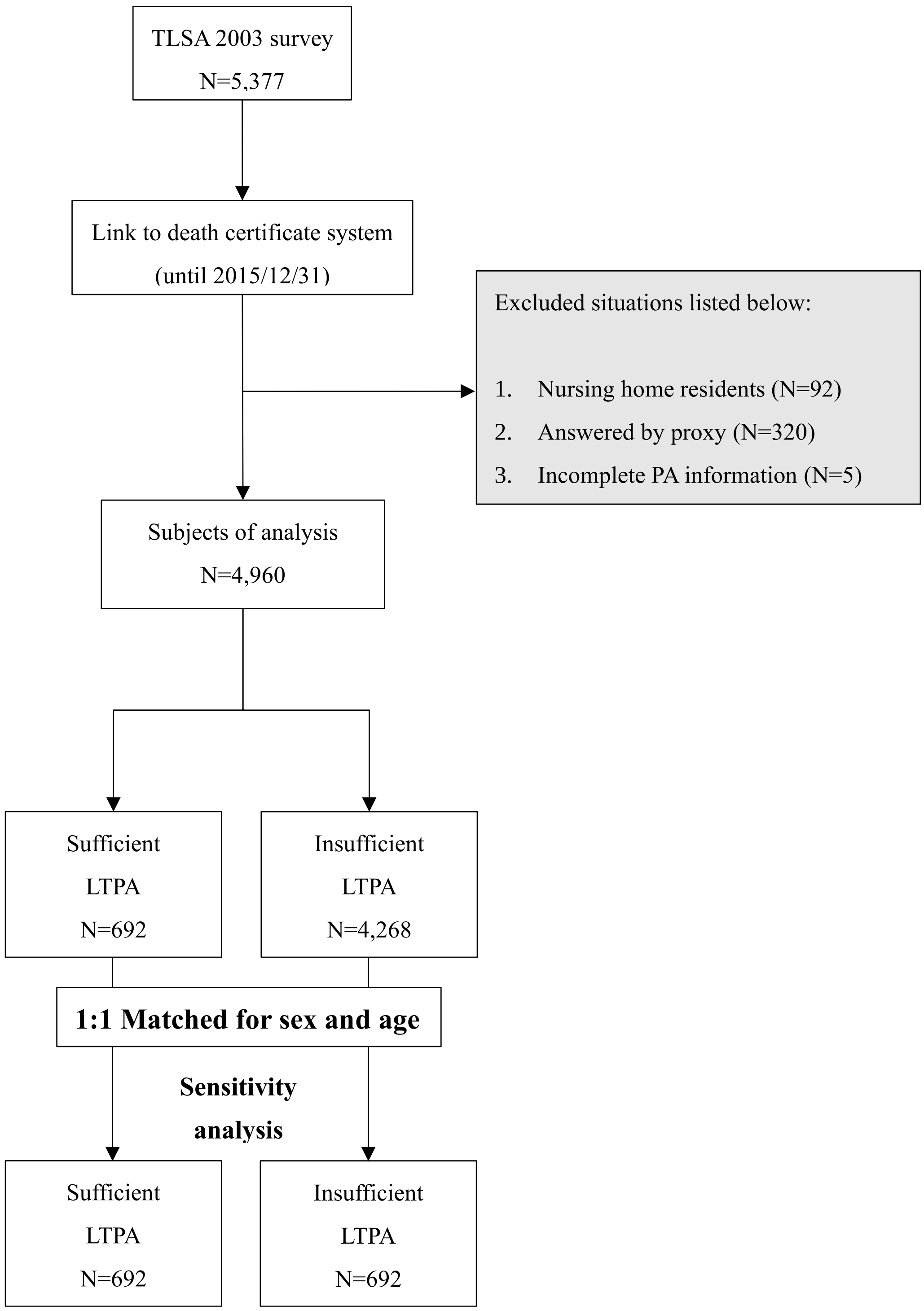

eFigure 3. Flow chart of inclusion and exclusion in 3rd sensitivity analyses. LTPA, leisure-time physical activity.

eTable 1. Baseline characteristics of participants with insufficient LTPA by the patterns of HPA, and TLSA, 2003

Characteristics		Type of HPA			*P*-value
		HPA, N (%)	No HPA, N (%)	Total, N	
		1,695 (39.71)	2,573 (60.29)	4,268	
Sex					
	male	348 (20.53)	1,798 (69.88)	2,146	<0.001
	female	1,347 (79.47)	775 (30.12)	2,122	
Age group, years					
	50–64	999 (58.94)	1,215 (47.22)	2,214	<0.001
	65–74	379 (22.36)	555 (21.57)	934	
	75–84	298 (17.58)	698 (27.13)	996	
	≥85	19 (1.12)	105 (4.08)	124	
Educational level					
	Without formal education	478 (28.2)	617 (23.98)	1,095	<0.001
	Elementary school	754 (44.48)	1,081 (42.01)	1,835	
	Junior to senior high school	347 (20.47)	612 (23.79)	959	
	College degree and above	116 (6.84)	263 (10.22)	379	
Marital status					
	Married	1,074 (63.36)	1,974 (76.72)	3,048	<0.001
	Unmarried	621 (36.64)	599 (23.28)	1,220	
Area					
	Rural	556 (32.8)	885 (34.4)	1,441	0.138
	Suburban	378 (22.3)	623 (24.21)	1,001	
	Urban	756 (44.6)	1,056 (41.04)	1,812	
	*missing	5 (0.29)	9 (0.35)	14	
Economic satisfaction					
	Satisfied	570 (33.63)	1044 (40.58)	1614	<0.001
	Fair	581 (34.28)	879 (34.16)	1460	
	Dissatisfied	544 (32.09)	650 (25.26)	1194	
BMI, kg/m^2					
	Underweight	88 (5.19)	123 (4.78)	211	0.095

	Normal weight	795 (46.9)	1,187 (46.13)	1,982	
	Overweight	440 (25.96)	721 (28.02)	1,161	
	Obesity	320 (18.88)	434 (16.87)	754	
	*missing	52 (3.07)	108 (4.2)	160	
Smoking status					
	Never	1,387 (81.83)	1,285 (49.94)	2,672	<0.001
	Former	105 (6.19)	548 (21.3)	653	
	Current	203 (11.98)	740 (28.76)	943	
Alcohol intake					
	Lifetime abstainer	1,326 (78.23)	1,670 (64.9)	2,996	<0.001
	Infrequent	134 (7.91)	259 (10.07)	393	
	Light to moderate	164 (9.68)	432 (16.79)	596	
	Heavy	71 (4.19)	212 (8.24)	283	
Hypertension					
	No	1,181 (69.68)	1,735 (67.43)	2,916	0.123
	Yes	514 (30.32)	838 (32.57)	1,352	
Diabetes					
	No	1,512 (89.2)	2,169 (84.3)	3,681	<0.001
	Yes	183 (10.8)	404 (15.7)	587	
Heart disease					
	No	1,431 (84.42)	2,161 (83.99)	3,592	0.702
	Yes	264 (15.58)	412 (16.01)	676	
Stroke					
	No	1,666 (98.29)	2,423 (94.17)	4,089	<0.001
	Yes	29 (1.71)	150 (5.83)	179	
Cancer					
	No	1,654 (97.58)	2,489 (96.74)	4,143	0.109
	Yes	41 (2.42)	84 (3.26)	125	

HPA, household physical activity; LTPA, leisure-time physical activity; TLSA, Taiwan Longitudinal Study on Aging.

eTable 2. Baseline characteristics of participants by the patterns of LTPA in 1st sensitivity analysis, TLSA, 2003

Characteristics	Type of LTPA			*P*-value
	Sufficient LTPA, N (%)	Insufficient LTPA, N (%)	Total, N	
	676 (14.06)	4,132 (85.94)	4,808	
Sex				
male	401 (59.32)	2,079 (50.31)	2,480	<0.001
female	275 (40.68)	2,053 (49.69)	2,328	
Age group, years				
50–64	343 (50.74)	2,213 (53.56)	2,556	0.184
65–74	173 (25.59)	928 (22.46)	1,101	
75–84	160 (23.67)	991 (23.98)	1,151	
Educational level				
Without formal education	136 (20.12)	1,016 (24.59)	1,152	0.027
Elementary school	293 (43.34)	1,799 (43.54)	2,092	
Junior to senior high school	172 (25.44)	944 (22.85)	1,116	
College degree and above	75 (11.09)	373 (9.03)	448	
Marital status				
Married	500 (73.96)	3,005 (72.73)	3,505	0.502
Unmarried	176 (26.04)	1,127 (27.27)	1,303	
Area				
Rural	209 (30.92)	1,395 (33.76)	1,604	0.038
Suburban	139 (20.56)	967 (23.4)	1,106	
Urban	327 (48.37)	1,758 (42.55)	2,085	
*missing	1 (0.15)	12 (0.29)	13	
Economic satisfaction				
Satisfied	283 (41.86)	1,559 (37.73)	1,842	0.012
Fair	238 (35.21)	1,405 (34)	1,643	
Dissatisfied	155 (22.93)	1,168 (28.27)	1,323	
BMI, kg/m^2				
Underweight	25 (3.7)	198 (4.79)	223	0.097

	Normal weight	315 (46.6)	1,914 (46.32)	2,229	
	Overweight	190 (28.11)	1,136 (27.49)	1,326	
	Obesity	134 (19.82)	741 (17.93)	875	
	*missing	12 (1.78)	143 (3.46)	155	
Smoking status					
	Never	368 (54.44)	2,588 (62.63)	2,956	<0.001
	Former	149 (22.04)	620 (15)	769	
	Current	159 (23.52)	924 (22.36)	1,083	
Alcohol intake					
	Lifetime abstainer	456 (67.46)	2,879 (69.68)	3,335	0.253
	Infrequent	80 (11.83)	387 (9.37)	467	
	Light to moderate	96 (14.2)	591 (14.3)	687	
	Heavy	44 (6.51)	275 (6.66)	319	
Hypertension					
	No	416 (61.54)	2,825 (68.37)	3,241	<0.001
	Yes	260 (38.46)	1,307 (31.63)	1,567	
Diabetes					
	No	583 (86.24)	3,561 (86.18)	4,144	0.966
	Yes	93 (13.76)	571 (13.82)	664	
Heart disease					
	No	545 (80.62)	3,491 (84.49)	4,036	0.011
	Yes	131 (19.38)	641 (15.51)	772	
Stroke					
	No	647 (95.71)	3,963 (95.91)	4,610	0.808
	Yes	29 (4.29)	169 (4.09)	198	
Cancer					
	No	661 (97.78)	4,013 (97.12)	4,674	0.333
	Yes	15 (2.22)	119 (2.88)	134	

LTPA, leisure-time physical activity; TLSA, Taiwan Longitudinal Study on Aging.

eTable 3. Baseline characteristics of participants by the patterns of LTPA in 2nd sensitivity analysis, TLSA, 2003

Characteristics		Type of LTPA			*P*-value
		Sufficient LTPA, N (%) 322 (12.10)	Insufficient LTPA, N (%) 2,339 (87.90)	Total, N 2,661	
Sex					
	male	194 (60.25)	1,248 (53.36)	1,442	0.020
	female	128 (39.75)	1,091 (46.64)	1,219	
Age group, years					
	50–64	197 (61.18)	1,483 (63.4)	1,680	0.267
	65–74	69 (21.43)	400 (17.1)	469	
	75–84	49 (15.22)	393 (16.8)	442	
	≥85	7 (2.17)	63 (2.69)	70	
Educational level					
	Without formal education	57 (17.7)	494 (21.12)	551	0.087
	Elementary school	137 (42.55)	1,050 (44.89)	1,187	
	Junior to senior high school	83 (25.78)	561 (23.98)	644	
	College degree and above	45 (13.98)	234 (10)	279	
Marital status					
	Married	245 (76.09)	1,761 (75.29)	2,006	0.755
	Unmarried	77 (23.91)	578 (24.71)	655	
Area					
	Rural	96 (29.81)	839 (35.87)	935	0.080
	Suburban	70 (21.74)	537 (22.96)	607	
	Urban	155 (48.14)	955 (40.83)	1,110	
	*missing	1 (0.31)	8 (0.34)	9	
Economic satisfaction					
	Satisfied	145 (45.03)	875 (37.41)	1,020	0.008
	Fair	111 (34.47)	816 (34.89)	927	
	Dissatisfied	66 (20.5)	648 (27.7)	714	
BMI, kg/m^2					

	Underweight	12 (3.73)	111 (4.75)	123	0.656
	Normal weight	167 (51.86)	1,221 (52.2)	1,388	
	Overweight	93 (28.88)	610 (26.08)	703	
	Obesity	43 (13.35)	323 (13.81)	366	
	*missing	7 (2.17)	74 (3.16)	81	
Smoking status					
	Never	181 (56.21)	1,430 (61.14)	1,611	0.014
	Former	60 (18.63)	298 (12.74)	358	
	Current	81 (25.16)	611 (26.12)	692	
Alcohol intake					
	Lifetime abstainer	193 (59.94)	1,524 (65.16)	1,717	0.135
	Infrequent	46 (14.29)	242 (10.35)	288	
	Light to moderate	56 (17.39)	397 (16.97)	453	
	Heavy	27 (8.39)	176 (7.52)	203	

LTPA, leisure-time physical activity; TLSA, Taiwan Longitudinal Study on Aging.

eTable 4. Baseline characteristics of matched participants by the patterns of LTPA in 3rd sensitivity analysis, TLSA, 2003

	Type of LTPA			*P*-value
Characteristics	Sufficient LTPA, N (%) 692 (50)	Insufficient LTPA, N (%) 692 (50)	Total, N 1,384	
Sex				
male	410 (59.25)	410 (59.25)	820	1
female	282 (40.75)	282 (40.75)	564	
Age group, years				
50–64	344 (49.71)	344 (49.71)	688	1
65–74	174 (25.14)	174 (25.14)	348	
75–84	161 (23.27)	161 (23.27)	322	
≥85	13 (1.88)	13 (1.88)	26	
Educational level				
Without formal education	142 (20.52)	163 (23.55)	305	0.421
Elementary school	300 (43.35)	303 (43.79)	603	
Junior to senior high school	173 (25)	160 (23.12)	333	
College degree and above	77 (11.13)	66 (9.54)	143	
Marital status				
Married	509 (73.55)	508 (73.41)	1,017	0.951
Unmarried	183 (26.45)	184 (26.59)	367	
Area				
Rural	217 (31.36)	238 (34.39)	455	0.037
Suburban	139 (20.09)	170 (24.57)	309	
Urban	335 (48.41)	283 (40.9)	618	
*missing	1 (0.14)	1 (0.14)	2	
Economic satisfaction				
Satisfied	291 (42.05)	267 (38.58)	558	0.307
Fair	243 (35.12)	246 (35.55)	489	
Dissatisfied	158 (22.83)	179 (25.87)	337	
BMI, kg/m^2				

	Underweight	27 (3.9)	27 (3.9)	54	0.275
	Normal weight	324 (46.82)	354 (51.16)	678	
	Overweight	193 (27.89)	176 (25.43)	369	
	Obesity	134 (19.36)	114 (16.47)	248	
	*missing	14 (2.02)	21 (3.03)	35	
Smoking status					
	Never	378 (54.62)	384 (55.49)	762	0.057
	Former	152 (21.97)	120 (17.34)	272	
	Current	162 (23.41)	188 (27.17)	350	
Alcohol intake					
	Lifetime abstainer	468 (67.63)	477 (68.93)	945	0.55
	Infrequent	80 (11.56)	64 (9.25)	144	
	Light to moderate	97 (14.02)	104 (15.03)	201	
	Heavy	47 (6.79)	47 (6.79)	94	
Hypertension					
	No	428 (61.85)	475 (68.64)	903	0.008
	Yes	264 (38.15)	217 (31.36)	481	
Diabetes					
	No	596 (86.13)	605 (87.43)	1,201	0.475
	Yes	96 (13.87)	87 (12.57)	183	
Heart disease					
	No	559 (80.78)	578 (83.53)	1,137	0.182
	Yes	133 (19.22)	114 (16.47)	247	
Stroke					
	No	663 (95.81)	665 (96.1)	1,328	0.785
	Yes	29 (4.19)	27 (3.9)	56	
Cancer					
	No	676 (97.69)	673 (97.25)	1,349	0.608
	Yes	16 (2.31)	19 (2.75)	35	

LTPA, leisure-time physical activity; TLSA, Taiwan Longitudinal Study on Aging.

eTable 5. Baseline characteristics of participants with insufficient LTPA by the patterns of HPA in 1st sensitivity analysis, TLSA, 2003

Characteristics	Type of HPA			*P*-value
	HPA, N (%)	No HPA, N (%)	Total, N	
	1,674 (40.51)	2,458 (59.49)	4,132	
Sex				
male	340 (20.31)	1,739 (70.75)	2,079	<0.001
female	1,334 (79.69)	719 (29.25)	2,053	
Age group, years				
50–64	999 (59.68)	1,214 (49.39)	2,213	<0.001
65–74	378 (22.58)	550 (22.38)	928	
75–84	297 (17.74)	694 (28.23)	991	
Educational level				
Without formal education	466 (27.84)	550 (22.38)	1,016	<0.001
Elementary school	748 (44.68)	1,051 (42.76)	1,799	
Junior to senior high school	345 (20.61)	599 (24.37)	944	
College degree and above	115 (6.87)	258 (10.5)	373	
Marital status				
Married	1,071 (63.98)	1,934 (78.68)	3,005	<0.001
Unmarried	603 (36.02)	524 (21.32)	1,127	
Area				
Rural	548 (32.74)	847 (34.46)	1,395	0.088
Suburban	371 (22.16)	596 (24.25)	967	
Urban	751 (44.86)	1,007 (40.97)	1,758	
*missing	4 (0.24)	8 (0.33)	12	
Economic satisfaction				
Satisfied	566 (33.81)	993 (40.4)	1,559	<0.001
Fair	570 (34.05)	835 (33.97)	1,405	
Dissatisfied	538 (32.14)	630 (25.63)	1,168	
BMI, kg/m^2				
Underweight	86 (5.14)	112 (4.56)	198	0.229
Normal weight	784 (46.83)	1,130 (45.97)	1,914	

Overweight	437 (26.11)	699 (28.44)	1,136	
Obesity	316 (18.88)	425 (17.29)	741	
*missing	51 (3.05)	92 (3.74)	143	
Smoking status				
Never	1,371 (81.9)	1,217 (49.51)	2,588	<0.001
Former	102 (6.09)	518 (21.07)	620	
Current	201 (12.01)	723 (29.41)	924	
Alcohol intake				
Lifetime abstainer	1,307 (78.08)	1,572 (63.95)	2,879	<0.001
Infrequent	134 (8)	253 (10.29)	387	
Light to moderate	163 (9.74)	428 (17.41)	591	
Heavy	70 (4.18)	205 (8.34)	275	
Hypertension				
No	1,168 (69.77)	1,657 (67.41)	2,825	0.109
Yes	506 (30.23)	801 (32.59)	1,307	
Diabetes				
No	1,492 (89.13)	2,069 (84.17)	3,561	<0.001
Yes	182 (10.87)	389 (15.83)	571	
Heart disease				
No	1,414 (84.47)	2,077 (84.5)	3,491	0.978
Yes	260 (15.53)	381 (15.5)	641	
Stroke				
No	1,647 (98.39)	2,316 (94.22)	3,963	<0.001
Yes	27 (1.61)	142 (5.78)	169	
Cancer				
No	1,633 (97.55)	2,380 (96.83)	4,013	0.172
Yes	41 (2.45)	78 (3.17)	119	

HPA, household physical activity; LTPA, leisure-time physical activity; TLSA, Taiwan Longitudinal Study on Aging.

eTable 6. Baseline characteristics of participants with insufficient LTPA by the patterns of HPA in 2nd sensitivity analysis, TLSA, 2003

Characteristics		Type of HPA			*P*-value
		HPA, N (%)	No HPA, N (%)	Total, N	
		972 (41.56)	1,367 (58.44)	2,339	
Sex					
	male	200 (20.58)	1,048 (76.66)	1,248	<0.001
	female	772 (79.42)	319 (23.34)	1,091	
Age group, years					
	50–64	661 (68)	822 (60.13)	1,483	<0.001
	65–74	176 (18.11)	224 (16.39)	400	
	75–84	124 (12.76)	269 (19.68)	393	
	≥85	11 (1.13)	52 (3.8)	63	
Educational level					
	Without formal education	215 (22.12)	279 (20.41)	494	0.037
	Elementary school	454 (46.71)	596 (43.6)	1,050	
	Junior to senior high school	224 (23.05)	337 (24.65)	561	
	College degree and above	79 (8.13)	155 (11.34)	234	
Marital status					
	Married	643 (66.15)	1,118 (81.78)	1,761	<0.001
	Unmarried	329 (33.85)	249 (18.22)	578	
Area					
	Rural	324 (33.33)	515 (37.67)	839	0.004
	Suburban	207 (21.3)	330 (24.14)	537	
	Urban	439 (45.16)	516 (37.75)	955	
	*missing	2 (0.21)	6 (0.44)	8	
Economic satisfaction					
	Satisfied	325 (33.44)	550 (40.23)	875	<0.001
	Fair	341 (35.08)	475 (34.75)	816	
	Dissatisfied	306 (31.48)	342 (25.02)	648	
BMI, kg/m^2					
	Underweight	49 (5.04)	62 (4.54)	111	0.010

Normal weight	530 (54.53)	691 (50.55)	1,221	
Overweight	222 (22.84)	388 (28.38)	610	
Obesity	147 (15.12)	176 (12.87)	323	
*missing	24 (2.47)	50 (3.66)	74	
Smoking status				
Never	797 (82)	633 (46.31)	1,430	<0.001
Former	56 (5.76)	242 (17.7)	298	
Current	119 (12.24)	492 (35.99)	611	
Alcohol intake				
Lifetime abstainer	734 (75.51)	790 (57.79)	1,524	<0.001
Infrequent	82 (8.44)	160 (11.7)	242	
Light to moderate	111 (11.42)	286 (20.92)	397	
Heavy	45 (4.63)	131 (9.58)	176	

HPA, household physical activity; LTPA, leisure-time physical activity; TLSA, Taiwan Longitudinal Study on Aging.

eTable 7. Wald Tests of HPA*LTPA Effect

HPA*LTPA Effects		
Wald Chi-Square	Pr > ChiSq	Model
5.8684	0.0154	Model 1[a]
4.6454	0.0311	Model 2[b]
2.3926	0.1219	Model 3[c]

HPA, household physical activity; LTPA, leisure-time physical activity.

[a]Model 1: Adjusted for sex, age

[b]Model 2: model 1 + educational, marital, area, economic satisfaction, body mass index, smoking status, and alcohol intake

[c]Model 3: model 2 + chronic health conditions

eTable 8, Association of the patterns of combined LTPA and HPA with subsequent risk of all-cause mortality (N=4,960)

Groups	N	Number of deaths	Hazard ratio (95% confidence interval)	*P*-value
Model 1[a]				
Insufficient LTPA and No HPA	2,573	1,057	1 [Reference]	
Insufficient LTPA and HPA	1,695	436	0.79 (0.7–0.89)	<0.001
Sufficient LTPA and No HPA	440	146	0.71 (0.6–0.85)	<0.001
Sufficient LTPA and HPA	252	73	0.82 (0.64–1.04)	0.104
Model 2[b]				
Insufficient LTPA and No HPA	2,573	1,057	1 [Reference]	
Insufficient LTPA and HPA	1,695	436	0.79 (0.7–0.89)	0.001
Sufficient LTPA and No HPA	440	146	0.75 (0.63–0.89)	<0.001
Sufficient LTPA and HPA	252	73	0.82 (0.65–1.05)	0.111
Model 3[c]				
Insufficient LTPA and No HPA	2,573	1,057	1 [Reference]	
Insufficient LTPA and HPA	1,695	436	0.85 (0.76–0.96)	0.010
Sufficient LTPA and No HPA	440	146	0.78 (0.65–0.93)	0.005
Sufficient LTPA and HPA	252	73	0.84 (0.66–1.08)	0.170

HPA, household physical activity; LTPA, leisure-time physical activity.

[a]Model 1: Adjusted for sex and age

[b]Model 2: model 1 + educational, marital, area, economic satisfaction, body mass index, smoking status, and alcohol intake

[c]Model 3: model 2 + chronic health conditions

eTable 9. Subgroup analysis of the association of the patterns of combined LTPA and HPA with subsequent risk of all-cause mortality

	Men		Women	
	Hazard ratios (95% confidence intervals)	P-value	Hazard ratios (95% confidence intervals)	*P*-value
Model 1[a]				
Insufficient LTPA and No HPA	1 [Reference]		1 [Reference]	
Insufficient LTPA and HPA	1.21 (1.02–1.42)	0.027	0.57 (0.48–0.67)	<0.001
Sufficient LTPA and No HPA	0.87 (0.72–1.06)	0.16	0.37 (0.24–0.58)	<0.001
Sufficient LTPA and HPA	1.04 (0.7–1.55)	0.845	0.64 (0.48–0.87)	0.005
Model 2[b]				
Insufficient LTPA and No HPA	1 [Reference]		1 [Reference]	
Insufficient LTPA and HPA	1.15 (0.96–1.37)	0.14	0.61 (0.51–0.72)	<0.001
Sufficient LTPA and No HPA	0.93 (0.76–1.12)	0.431	0.37 (0.24–0.58)	<0.001
Sufficient LTPA and HPA	0.98 (0.65–1.48)	0.932	0.69 (0.51–0.94)	0.017
Model 3[c]				
Insufficient LTPA and No HPA	1 [Reference]		1 [Reference]	
Insufficient LTPA and HPA	1.17 (0.98–1.41)	0.091	0.7 (0.59–0.83)	<0.001
Sufficient LTPA and No HPA	0.95 (0.78–1.15)	0.606	0.38 (0.24–0.59)	<0.001
Sufficient LTPA and HPA	1.01 (0.67–1.51)	0.975	0.71 (0.53–0.97)	0.031

HPA, household physical activity; LTPA, leisure-time physical activity.

[a]Model 1: Adjusted for age

[b]Model 2: model 1 + educational, marital, area, economic satisfaction, body mass index, smoking status, and alcohol intake

[c]Model 3: model 2 + chronic health conditions

eTable 10. Association between HPA patterns and subsequent risk of all-cause mortality among overall participants (N=4,960)

Group	N	Number of deaths	Person-years	Incidence rate (/10^3 person-year)	Hazard ratio (95% confidence interval)	*P*-value	Model
No HPA	3,013	1,203	29,610	40.1	1 [Reference]		
HPA	1,947	509	20,897	24.4	0.83 (0.74–0.93)	0.001	Model 1[a]
					0.82 (0.73–0.92)	<0.001	Model 2[b]
					0.88 (0.79–0.99)	0.028	Model 3[c]

HPA, household physical activity.

[a]Model 1: Adjusted for sex and age

[b]Model 2: model 1 + educational, marital, area, economic satisfaction, body mass index, smoking status, and alcohol intake

[c]Model 3: model 2 + chronic health conditions

eTable 11. Association between HPA patterns and subsequent risk of all-cause mortality among people with sufficient LTPA (N=692)

Group	N	Number of deaths	Person-years	Incidence rate (/10^3 person-year)	Hazard ratio (95% confidence interval)	*P*-value	Model
No HPA	440	146	4,525	32.3	1 [Reference]		
HPA	252	73	2,673	27.3	1.35 (0.97–1.86)	0.072	Model 1[a]
					1.21 (0.87–1.68)	0.268	Model 2[b]
					1.22 (0.87–1.70)	0.249	Model 3[c]

HPA, household physical activity; LTPA, leisure-time physical activity.

[a]Model 1: Adjusted for sex and age

[b]Model 2: model 1 + educational, marital, area, economic satisfaction, body mass index, smoking status, and alcohol intake

[c]Model 3: model 2 + chronic health conditions
